# German Shorthaired Pointer dogs with exfoliative cutaneous lupus erythematosus develop immune-complex membranous glomerulonephropathy

**DOI:** 10.1177/03009858231173362

**Published:** 2023-05-24

**Authors:** Hayley K. Amerman, Rachel E. Cianciolo, Margret L. Casal, Elizabeth Mauldin

**Affiliations:** 1 University of Pennsylvania, Philadelphia, PA; 2 The Ohio State University, Columbus, OH

**Keywords:** canine, cutaneous lupus erythematosus, glomerular disease, immune-complex glomerulopathy, proteinuria, renal biopsy

## Abstract

German Shorthaired Pointer (GSHP) dogs with a *UNC93B1* gene mutation develop exfoliative cutaneous lupus erythematosus (ECLE) and kidney disease resembling lupus nephritis in humans. The objective of this study was to characterize the kidney disease by light microscopy, immunofluorescence, and electron microscopy in a population of GSHP dogs with ECLE. Medical records were reviewed, and light microscopy of kidneys from 7 GSHP dogs with a previous histologic diagnosis of ECLE was performed. Immunofluorescence of fresh-frozen kidney from 1 dog and transmission electron microscopy of kidney from that dog and 2 additional dogs were performed. Five of 7 dogs had proteinuria diagnosed by urinalysis or urine protein-to-creatinine ratio. Two of 7 dogs were intermittently hypoalbuminemic, and none were azotemic. Histologic findings included early (2 dogs) to late (5 dogs) membranous glomerulonephropathy characterized by mild-to-severe glomerular capillary loop thickening and tubular proteinosis. In all 7 cases, trichrome staining revealed red granular immune deposits on the subepithelial surface of the glomerular basement membrane. Immunofluorescence revealed strong granular labeling for immunoglobulins and complement protein C3. Electron microscopy demonstrated subepithelial electron-dense immune deposits encircled by the remodeled glomerular basement membrane. These findings are diagnostic of immune-complex membranous glomerulonephropathy and are similar to class V lupus in humans. This cohort of GSHP dogs with ECLE developed immune-complex membranous glomerulonephropathy, which we hypothesize is a manifestation of systemic lupus erythematosus. GSHP dogs with ECLE should undergo clinical evaluation of renal function for early identification and treatment.

Cutaneous lupus erythematosus (CLE) in dogs is an uncommon, acquired disorder with a variety of manifestations. The most common form is discoid lupus erythematosus that is usually confined to the nasal planum. Other breed-specific forms include vesicular CLE described in several collie-related breeds and exfoliative CLE (ECLE).^12,14^ Systemic lupus erythematosus (SLE) in dogs is rare and generally manifests as glomerulonephritis, fever, nonerosive polyarthritis, and/or hematologic disorders (e.g., hemolytic anemia, thrombocytopenia, and lymphopenia).^16^ The affected dogs can have nonspecific skin reactions, such as pyoderma or vasculitis, but cutaneous lupus-specific reactions, characterized by a lymphocytic interface dermatitis with basal keratinocyte apoptosis, are rare.^12^ In general, dogs with CLE do not progress to develop SLE.

German Shorthaired Pointer (GSHP) dogs with a protein-altering, single base-pair variant in *UNC93B1* develop juvenile-onset ECLE, a debilitating and life-limiting skin condition that is initially characterized clinically by scaling and erythema that eventually progresses to pigment loss, erosions, and ulcers.^4,10,11,18^ These lesions develop on the muzzle and progress to involve the trunk, limbs, and ears. The dogs also develop a systemic disease, characterized by polyarthralgia, peripheral lymphadenopathy, hematologic abnormalities (mild thrombocytopenia, lymphopenia, and hyperproteinemia), and infertility. The dermatologic manifestation can be debilitating, refractory to medical management, and is often the reason for euthanasia.^8,11^ The hypothesis of this study is that this subset of GSHP dogs with the *UNC93B1* variant develop ECLE and immune-mediated kidney disease as a systemic manifestation of lupus, similar to that seen in humans. The goal of this study was to characterize the kidney disease by light microscopy, immunofluorescence, and transmission electron microscopy.

## Materials and Methods

**Figure 1.**
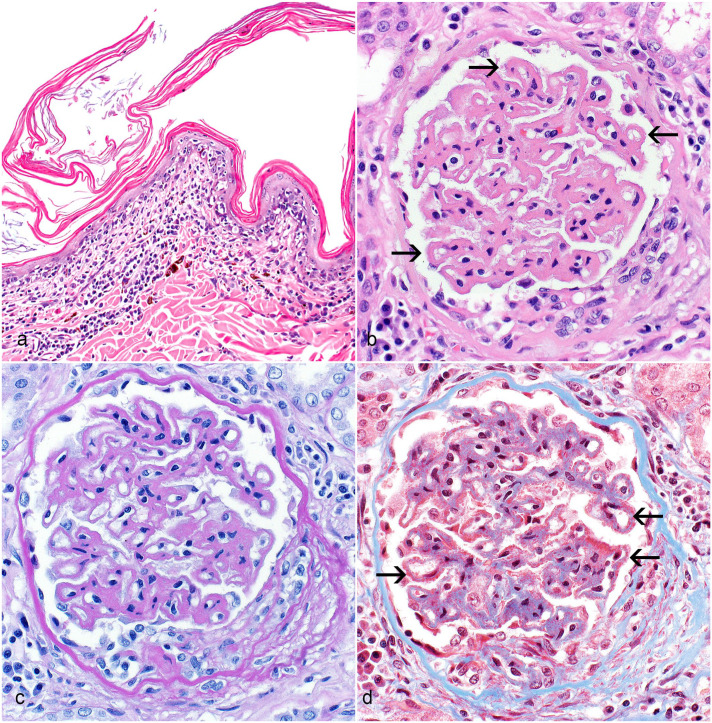
Histologic lesions in the skin and kidney of German Shorthair Pointer (GSHP) dogs with exfoliative cutaneous lupus erythematosus. (a) Exfoliative cutaneous lupus erythematosus, haired skin, 10-month-old GSHP dog. The epidermal changes are characterized by lymphocytic interface dermatitis with basal layer vacuolar change, pigmentary incontinence, and orthokeratotic hyperkeratosis. Hematoxylin and eosin (HE). (b–d) Late membranous glomerulonephropathy, kidney, dog. (b, c) Glomeruli are characterized by global diffuse capillary loop thickening (arrows), mild mesangial hypercellularity, Bowman’s capsule thickening, and synechia. HE (b) and period acid-Schiff (c). (d) Numerous small red granular immune deposits are on the external (subepithelial) surface of the glomerular basement membrane (arrows). Masson’s trichrome.

Seven cases were included in this cohort using the following inclusion criteria: (1) GSHP dog, (2) a previous clinical and histologic diagnosis of ECLE (Fig. 1a), (3) available formalin-fixed paraffin-embedded kidney tissue, and (4) a complete blood count and serum biochemistry for review. Urinalysis results and urine protein-to-creatinine ratios (UPC) were also reviewed when available. Five of the 7 dogs were related and part of a colony at the University of Pennsylvania. Archival tissue and medical records were reviewed from these 5 GSHPs previously housed at PennVet via an approved protocol. Two dogs were client-owned, and pedigree analysis revealed that they were distantly related to the colony dogs.

### Light Microscopy

All specimens for light microscopy evaluation were immersion fixed in 10% buffered formalin, processed, and embedded in paraffin. Tissues were serially sectioned at a thickness of 3 µm and stained with hematoxylin and eosin, periodic acid–Schiff reagent, and Masson trichrome. All histochemical procedures were performed using established methods.^5,6^

### Electron Microscopy

Renal tissue was retrieved from formalin-fixed, paraffin-embedded blocks of kidney from 3 dogs. A portion of tissue was cored from the formalin-fixed, paraffin-embedded block, and excess paraffin was removed by applying gentle heat followed by xylene, hydration through an ethanol series, and washing several times in deionized water before postfixation. All samples were postfixed in 1% osmium tetroxide, serially dehydrated in graded alcohols, infiltrated with acetone (or propylene oxide) and epoxy plastic, and embedded in plastic. Sections measuring 1 µm in thickness were cut and stained with toluidine blue to locate glomeruli before thin sections were cut at 60 nm using an ultramicrotome (Ultracut S; Reichert Technologies, Depew, NY), placed on copper slot grids coated with Formvar (Electron Microscopy Services, Hatfield, PA), and stained with uranyl acetate and lead citrate. The grids were examined under a transmission electron microscope (JEOL JEM-1210 120Kv, JEOL Ltd.), and multiple digital images were acquired (REC, AMT XR41C Bottom-Mount CCD Camera, Advanced Microscopy Techniques Corp, Woburn, MA).

### Immunofluorescence

The tissue samples were frozen fresh in plastic cryomolds filled with TissueTek OCT embedding compound (Electron Microscopy Sciences, Fort Washington, PA). Blocks were stored at −20°C until shipped. A tissue sample from 1 dog was thawed, washed 3 times in Michel’s Wash (Newcomer Supply, Middleton, WI), placed in a plastic cryomold filled with TissueTek OCT embedding compound, and snap-frozen in liquid nitrogen. The block was stored at −80°C until sectioned. Thin (4 µm) cryosections were cut on a Leica CM 1850 UV cryostat (Bannockburn, IL) and stored at −80°C until they were thawed for 1 hour at room temperature for immunolabeling. Sections were fixed for 5 minutes in cold 100% acetone, air-dried for 1 hour, and then rehydrated in phosphate-buffered saline. Direct immunofluorescence was performed with fluorescein isothiocyanate–conjugated polyclonal goat antidog immunoglobulin G (IgG), IgM, IgA, and C3 antibodies (Bethyl Labs, Montgomery, TX), as well as with fluorescein isothiocyanate–conjugated polyclonal rabbit antihuman C1q, kappa light-chain, and lambda light-chain antibodies (Dako North America, Carpinteria, CA). Sections were incubated for 1 hour with an appropriate dilution of each antibody and then washed with phosphate-buffered saline. Sections were coverslipped using a mounting medium that retarded fluorescence quenching (Prolong Gold; Invitrogen, Carlsbad, CA) and were examined using appropriate filters with an epifluorescence microscope (Olympus, Center Valley, PA). A positive control was run on a previous diagnostic case that was already documented as having immune-complex glomerulonephritis. Negative controls were not used.

## Results

### Clinical Data

Dogs 1, 3, and 6 were intermittently hypoalbuminemic, but no dogs were azotemic. Dogs 3, 6, and 7 demonstrated intermittent lymphopenia, and dogs 1 and 4 were consistently lymphopenic. Dogs 1–5 were intermittently thrombocytopenic, and dogs 1–4 and 7 had elevated globulins. Five dogs had urinalyses available for review, all 5 of which were proteinuric based on positive urine protein (3+) or a UPC >0.5. Five dogs had antinuclear antibody (ANA) testing performed, 4 of which were consistently negative. Dog 7 developed an equivocal titer (1:25) over the course of 2 years, with a titer of 1:100 two months before euthanasia (Supplemental Table S1).

### Light Microscopy

Light microscopy was performed on all 7 dogs. Two of the 7 dogs had early membranous glomerulonephropathy (MGN), and the remaining 5 had late MGN, all characterized by global diffuse glomerular capillary loop thickening and minimal mesangial hypercellularity (Fig. 1b–d). In both the early and late cases, there was minimal hypercellularity within the mesangium and capillary lumens, characterized by slightly increased numbers of cell nuclei within the mesangium and occasionally within capillary loops. In the late cases, capillary walls were thickened up to 3 microns, with the thickened glomerular basement membrane (GBM) resulting in a wire-looped appearance. In all 7 cases, the trichrome stain revealed small red granular material (immune deposits) on the external (subepithelial) surface of the GBM. In the late cases, podocytes were diffusely hypertrophied focally to globally, as were many parietal epithelial cells. Both these cell lineages were occasionally degenerative, characterized by the presence of cytoplasmic vacuoles and rare hyaline droplets. There were rare synechiae and segmental sclerosis of multiple glomeruli. These glomerular changes are diagnostic for MGN.

Multifocally within both the early and late cases, the renal interstitium was expanded by lymphocytes, plasma cells, macrophages, and neutrophils that surrounded glomeruli, veins, and tubules. Tubules ranged from normal to degenerative, characterized by dilation, loss of the apical brush border, cytoplasmic vacuolation, and increased numbers of individual pyknotic cells. Scattered tubules were lined by basophilic, karyomegalic epithelial cells indicative of regeneration. Fibrosis and tubular atrophy were minimal.

### Immunofluorescence

**Figure 2.**
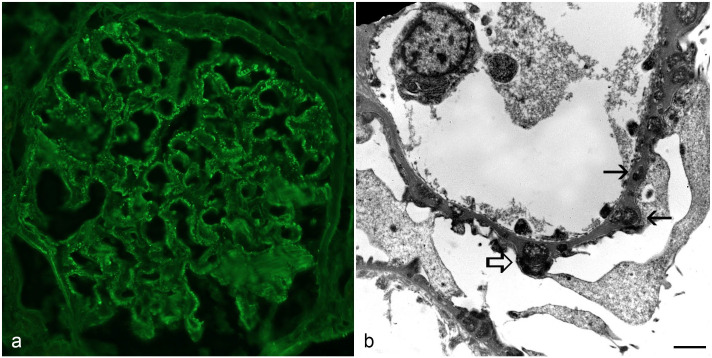
Late membranous glomerulonephropathy, kidney, dog. (a) Strong, granular IgG labeling is observed along glomerular basement membranes. IgG immunofluorescence. (b) Multiple electron-dense deposits are in the subepithelial zones of capillary loops and are encircled by new basement membrane material (solid arrows). Some have evidence of resorption with a mottled appearance (empty arrow). Transmission electron microscopy, scale bar = 1 μm.

Immunofluorescence was performed on fresh-frozen tissue samples from dog 6, which revealed strong granular labeling for lambda light chains, IgG heavy chains, and complement protein C3 (Fig. 2a).

### Transmission Electron Microscopy

Transmission electron microscopy was performed on formalin-fixed, paraffin-embedded blocks from dogs 5, 6, and 7, which revealed multiple electron-dense deposits along capillary walls, predominantly in subepithelial zones (Fig. 2b). One case was considered mild, with minimal-to-mild GBM remodeling characterized by multifocal slight thickening and disruption of the basement membrane architecture adjacent to immune-complex deposits, while 2 cases were considered severe in which the numerous immune-complex deposits were often completely encircled by new basement membrane material. Some deposits exhibited evidence of resorption, with a mottled appearance or an electron-lucent halo. The deposits varied in size from small to large with significant thickening of the GBM in the late cases. Occasionally, there were subendothelial deposits associated with subendothelial widening.

## Discussion

In humans, a diagnosis of SLE is based on the criteria established by the American College of Rheumatology and the European League Against Rheumatism.^2^ No single finding qualifies an individual as having SLE. Instead, 4 or more criteria (at least 1 clinical and 1 immunologic finding) must be met for a diagnosis of SLE.^2,7^ The criteria are routinely updated, and the current classification system is 90% effective in accurately diagnosing SLE. The 2019 criteria include (1) malar rash, (2) discoid rash, (3) photosensitivity, (4) oral/nasal ulcers, (5) polyarthritis, (6) serositis, (7) kidney disease evidenced by protein or casts, (8) certain neurological disorders (i.e., seizures, psychosis, delirium), and (9) hematologic abnormalities such as hemolytic anemia, leukopenia, and lymphopenia.^2,7^ The overwhelming majority of all people with systemic lupus have a positive ANA test, thus making it the most sensitive diagnostic test; however, a negative ANA test cannot rule out an SLE diagnosis.^2,7^ Up to 20% of patients may be ANA negative (true or false negative) at various stages of the disease (although typically the rate of ANA-negative systemic lupus is much lower).^7^

Chronic kidney disease associated with SLE is a significant cause of morbidity and mortality in human patients. The renal changes accompanied by proteinuria seen in this cohort of GSHP dogs resemble class V lupus nephritis in humans, defined as numerous glomerular capillary loop subepithelial immune deposits but no influx of leukocytes when evaluated by transmission electron microscopy.^1,3^ Furthermore, the dermatologic and hematologic changes seen in this cohort resemble those seen in human patients with SLE as well. Therefore, GSHP dogs with ECLE develop immune-complex glomerulonephritis that mimics progressive SLE in humans.

This is unique because SLE as a disease is rare in veterinary medicine, and SLE with concurrent lupus-specific dermatologic lesions (i.e., lymphocytic interface dermatitis, basal keratinocyte apoptosis) is rarer still. The diagnosis of SLE in veterinary species is made based on clinical signs, including polyarthritis, anemia, glomerulonephritis, thrombocytopenia, and dermatopathy, and a serum ANA titer.^16^ There are no firmly established criteria that must be met to diagnose SLE in veterinary patients as there are in human medicine, but it is generally accepted that 4 of 11 criteria established by the American College of Rheumatology must be met to diagnose SLE in veterinary species, as in humans. However, unlike in human medicine, the frequency of SLE-related skin disease is controversial. Although “skin disease” is considered a major sign of SLE, the precise lesions are often not well defined, and inclusion of nonspecific “skin disease” may erroneously fulfill the criteria for the diagnosis of SLE. Skin lesions that can be seen in veterinary patients with SLE are generally not histologically distinct for lupus (lupus nonspecific) and may be seen with other disease processes.^12^ Therefore, generalized skin lesions affecting multiple sites on an animal do not constitute a diagnosis of SLE.^12^ In Bichon Frisé, the diagnosis of type I bullous SLE was proposed because of the fulfillment of the following criteria: a diagnosis of SLE by standard methods; an acquired, vesicular, erosive, and ulcerative eruption; microscopic subepidermal vesicles with neutrophil-predominant inflammation at the dermoepidermal junction; IgG deposition at the epidermal basement membrane zone; and anti-type-VII collagen circulating IgG autoantibodies.^15^ Because this cohort of GSHP dogs develop a true lupus-specific skin disease as well as immune-complex glomerulonephritis, they may represent a unique hereditary form of SLE in veterinary medicine.

Given that there are significant differences between SLE in humans and ECLE in GSHP dogs, there are still several avenues left to explore to further define this disease process. Interestingly, 1 dog in this cohort had equivocal ANA titers, and the remaining dogs were all ANA negative. As in human medicine, there is no one particular clinical parameter used to diagnose SLE in veterinary medicine; as such, the sensitivity and specificity of ANA values in contributing to a diagnosis of SLE in dogs are dependent on the other criteria used to make the diagnosis. In 1 study evaluating ANA titers in a large group of dogs with and without SLE, of the 18 dogs with SLE, all 18 had ANA values greater than 1:160, which is compatible with seropositivity. In contrast, cross-classification of ANA titer by the number of major signs compatible with SLE revealed that most dogs seronegative for ANA had no or only 1 major sign compatible with a diagnosis of SLE.^16^ It is not clear why the patients in this cohort are not ANA seropositive, but it may be associated with the sporadic and/or intermittent nature of major signs in these dogs. Ongoing studies in this cohort will investigate if the dogs are positive for other antibodies to nuclear antigens that are often prevalent among many human autoimmune diseases, (e.g., anti–Sjögren’s-syndrome-related antigen A autoantibodies, or anti-Ro/SSA antibodies, as can be seen in SLE, neonatal lupus, and Sjogren’s syndrome in humans, as well as Sjogren’s-like syndrome in dogs).^13^

Given what is already known about the genetic variant in GSHP dogs, functional assays of the UNC93B1 protein and how it may affect Toll-like receptor 7 (TLR7) are needed to more accurately define their combined roles in this disease process. *UNC93B1* encodes the protein “unc-93 homolog B1, TLR signaling regulator,” a trafficking chaperone of TLR7, and thus is thought to mediate the correct trafficking and localization of TLRs from the endoplasmic reticulum to endosomes. Previous studies in humans and mice have shown that variants in similar genes causing complete loss of trafficking proteins result in immune deficiency, whereas altered expression can cause autoimmunity.^9,17^ The unc-93 homolog B1 protein normally interacts with TLR7 through a syndecan-binding protein, which dampens TLR7 signaling. The GSHP dogs with this gene variant have altered expression of the C-terminal tail of the UNC93B1 protein, and protein modeling studies predict that this altered expression may allow the UNC93B1 protein to interact with TLR7, but not syndecan binding protein.^10^ Therefore, because the variant leads to an amino acid substitution, it is possible that the normal interaction between TLR7 and UNC93B1 is not dampened, leading to the potential for development of autoimmunity. This relationship is yet to be defined in humans or mouse models, and thus, more scrutiny is warranted.

In conclusion, this cohort of GSHP dogs with ECLE developed immune-complex MGN, which we hypothesize is a manifestation of SLE in veterinary medicine and strongly suggests that GSHP dogs with ECLE should undergo clinical evaluation of renal function for early identification and treatment.

## Supplemental Material

sj-pdf-1-vet-10.1177_03009858231173362 – Supplemental material for German Shorthaired Pointer dogs with exfoliative cutaneous lupus erythematosus develop immune-complex membranous glomerulonephropathy Click here for additional data file. Supplemental material, sj-pdf-1-vet-10.1177_03009858231173362 for German Shorthaired Pointer dogs with exfoliative cutaneous lupus erythematosus develop immune-complex membranous glomerulonephropathy by Hayley K. Amerman, Rachel E. Cianciolo, Margret L. Casal and Elizabeth Mauldin in Veterinary Pathology
