# Supplementary material for: German Shorthaired Pointer dogs with exfoliative cutaneous lupus erythematosus develop immune-complex membranous glomerulonephropathy
Source: Vet Pathol. 2023 May 24;60(6):843–8. doi: 10.1177/03009858231173362 (PMC10583476; doi:10.1177/03009858231173362)
Supplement: sj-pdf-1-vet-10.1177_03009858231173362 – Supplemental material for German Shorthaired Pointer dogs with exfoliative cutaneous lupus erythematosus develop immune-complex membranous glomerulonephropathy [file sj-pdf-1-vet-10.1177_03009858231173362.pdf]

Supplemental Materials

German shorthaired pointer dogs with exfoliative cutaneous lupus erythematosus develop immune-complex membranous glomerulonephropathy

Hayley K. Amerman, Rachel E. Cianciolo, Margret L. Casal, Elizabeth Mauldin

Supplemental Table S1. Signalment and clinical findings in 7 German shorthaired pointer dogs with exfoliative cutaneous lupus erythematosus and membranous glomerulonephropathy

CASE NUMBER	AGE AT EUTHANASIA	SEX	URINALYSIS PROTEIN	URINE PROTEIN TO CREATININE RATIO	SIGNIFICANT SERUM CHEMISTRY FINDINGS	SIGNIFICANT CBC FINDINGS	ANA RESULTS
**1**	3 years 3 months	F	N/A	N/A	Intermittent hypoalbuminemia<br>Consistent hyperglobulinemia	Consistent lymphopenia<br>Intermittent pancytopenia	Negative at <1:40
**2**	1 year 4 months	M	3+	N/A	Hyperglobulinemia	Intermittent thrombocytopenia	N/A
**3**	4 years 6 months	M	3+	N/A	Intermittent hyperglobulinemia	Intermittent lymphopenia and thrombocytopenia	N/A
**4**	5 years 3 months	M	3+	N/A	Intermittent hyperglobulinemia and hypoalbuminemia	Consistent lymphopenia<br>Intermittent thrombocytopenia	N/A
**5**	1 year 3 months	M	3+	N/A	N/A	N/A	N/A
**6**	3 years 3 months	M	3+	Mean 13.2 +/- 4.9 (range 6.23-20.62)	Intermittent hypoalbuminemia	Intermittent lymphopenia<br>Intermittent thrombocytopenia	Negative at <1:40
**7**	3 years 4 months	M	N/A	N/A	Intermittent hyperglobulinemia	Intermittent lymphopenia	10/27/2008: Negative<br>6/16/2009: Positive @ 1:25<br>4/29/2010: Positive @ 1:100

F, female; M, male; N/A, not available
